# Nutrition integrated agricultural extension—a case study in Western Kenya

**DOI:** 10.1093/heapro/daab142

**Published:** 2021-09-07

**Authors:** Gudrun B Keding, Andreas Gramzow, Justus Ochieng, Alaik Laizer, Charity Muchoki, Charles Onyango, Peter Hanson, Ray-Yu Yang

**Affiliations:** 1 Center for Development Research, University of Bonn, Bonn, Germany Genscherallee 3, 53113 Bonn, Germany; 2 World Vegetable Center - Eastern and Southern Africa, Arusha, Tanzania P.O. Box 10, Duluti, Arusha, Tanzania; 3 World Vegetable Center - Eastern and Southern Africa, Arusha, Tanzania P.O. Box 10, Duluti, Arusha, Tanzania; 4 World Vegetable Center - Eastern and Southern Africa, Arusha, Tanzania P.O. Box 10, Duluti, Arusha, Tanzania; 5 National Commission for Science, Technology and Innovation, Nairobi, Kenya O. Box 30623, 00100 Nairobi, Kenya; 6 Syngenta Foundation for Sustainable Agriculture, 6th Floor, Avenue 5 Building, Rose Avenue, Nairobi, Kenya; 7 World Vegetable Center, West and Central Africa – Coastal and Humid Regions IITA-Benin Campus, 08 BP 0932 Tri Postal, Cotonou Benin; 8 World Vegetable Center, Headquarters, Tainan, No. 60, Yi-Min Liao, Shanhua, Tainan, 74199, Taiwan (ROC)

**Keywords:** Africa, communication, community-based intervention, nutrition, intersectoral partnerships

## Abstract

Integrating nutrition communication in agricultural intervention programs aimed at increased food availability and accessibility in resource-poor areas is crucial. To enhance the sustainability and scalability of nutrition communication, the present study piloted the approach of ‘nutrition integrated agricultural extension’ and tested nutrition-related outcomes with two types of nutrition messages (specific vs. sensitive) and two delivery channels (public sector vs. private sector). The study intervention comprised (i) vegetable seed kit distribution, (ii) ongoing agricultural extension activities by public or private sectors and (iii) nutrition communication with two different messages. The intervention was tested with three treatment arms and reached 454 farmers (>65% female) in rural Kakamega County, Western Kenya. Pre-/post-surveys measured outcome variables focused on farmers’ nutrition-related knowledge, attitudes and practices in vegetable production and consumption, and household dietary diversity score. Results showed that all treatments increased nutrition knowledge (*p* < 0.05). Nutrition-specific communication was more effective than nutrition-sensitive communication. Nutrition communication through either the public or the private agricultural sector was both effective. Before the study intervention, many participants believed that vegetable consumption was beneficial and wanted to increase intake. After the intervention, the number of participants who felt eating more vegetables was challenging decreased slightly. Nutrition communication was found to be especially important in conveying recommended food amounts and promoting increased vegetable consumption. Seasonality affected on-farm crop diversity and vegetable consumption results in this study.

## INTRODUCTION

Consuming diverse and nutritious diets helps to prevent malnutrition and reduce risks of non-communicable diseases (WHO, 2018). Yet, many resource-poor people are unaware of healthy diets and consume insufficient nutrient-rich food leading to micronutrient deficiencies (von Grebmer *et al.*, 2014). Nutrition knowledge, food environments and social, cultural and economic status are important determinants of dietary behaviors. To promote healthy eating in resource-poor areas, integrating nutrition communication in the intervention program aiming at enhanced food availability and accessibility is crucial (Wardle *et al.*, 2000; Dickson-Spillmann and Siegrist, 2011) and inclusion of smallholder farmers, both female and male in nutrition communication is highly recommended (Gómez *et al.*, 2011; FAO, 2013; Gillespie *et al.*, 2015).

It is well known that mothers’ knowledge of food choices can influence family dietary quality (Wardle *et al.*, 2000; Aguirre *et al.*, 2012). Nutrition education for mothers in improving young child nutrition and health is effective where good food is available (Penny *et al.*, 2005; Lartey, 2008). However, nutrition education for other household members and especially those responsible for food production has been less studied.

Traditional vegetables like amaranth leaves (*Amaranthus* spp.), African nightshades (*Solanum nigrum*, *Solanum* *scabrum* and *Solanum* *villosum*) and spider plant (*Gynandropsis gynandra*) contain high levels of vitamins, iron, calcium and proteins (Yang and Keding, 2009) that enrich diets and increase intakes of micronutrients (Weinberger and Pichop, 2009; Schreinemachers *et al.*, 2015). Vegetable supply chains in Sub-Saharan Africa are often short and smallholder farmers are both vegetable producers and consumers. Improved knowledge about the nutritional significance of crops and food diversity with traditional vegetables could influence decisions on the diversity of crops grown and foods consumed (Njoro *et al.*, 2013).

Delivery of nutrition messages has generally been considered the responsibility of public institutions, such as departments of health or education (schools) and sometimes augmented by non-government organizations (NGOs). However, government institutions are often under-funded and have difficulty reaching rural populations; NGOs vary in size, types of outreach and funding sustainability. Consequently, if nutrition messages could be in addition communicated by further institutions this could potentially increase the dissemination of nutritional knowledge. Many seed companies and other private input suppliers market seed and other products and services to farmers, often establishing extensive distribution channels in rural areas. Besides marketing crop varieties, some vegetable seed companies have technical advisors that train farmers in crop production practices so farmers benefit from the use of improved varieties and other inputs. While nutrition promotion is not the mission of seed companies, increased consumer demand and consumption of vegetables would lead to increased production and farmer seed purchases. Seed companies could be motivated to spread nutrition messages that lead to increased demand for vegetables. At the same time, seed and agribusiness companies as a vehicle for nutrition education need to be viewed with caution as with the profit motive they naturally place increased importance on rising sales of seeds and other inputs (Flachs and Stone, 2019) possibly at the expense of accurate nutrition information. Still, it was decided to test a local vegetable seed company against a public institution/NGO in delivering nutrition messages to small-scale farmers.

There are two general approaches to nutrition interventions, namely ‘nutrition-specific’ and ‘nutrition-sensitive’. A nutrition-specific intervention addresses the immediate causes of malnutrition, such as inadequate nutrient and dietary intakes, food-, water-, vector-borne and infectious diseases, and some underlying causes, such as poor childcare and poor access to hygiene and health services. A nutrition-sensitive intervention also addresses the underlying and basic causes of malnutrition by incorporating nutrition goals into the actions of other sectors, such as agriculture or education (Ruel and Alderman, 2013). Nutrition messages and way of communication can be designed based on the two approaches while at the same time being tailored to the local context is highly important (Kumar *et al.*, 2018).

This study aimed to test the effects of different nutrition messages using two delivery channels. Nutrition messages were integrated into ongoing agricultural extension training of local government and a seed company. The effects were tested on vegetable farmers in western Kenya by measuring nutrition-related knowledge, attitudes and practices (KAP).

### A short history of agricultural extension

The agricultural extension can be described as support to farmers in their endeavors to produce food and feed in the face of a changing environment (Knorr *et al.*, 2007). The (documented) history of advising farmers reaches back to around 1740 when the potato late blight epidemic in Ireland resulted in extreme famine and the viceroy urged local agricultural societies to advise impoverished smallholder farmers on ways to diversify their production system. Only in the mid-19th century ‘extension’ came up as a term when university lectures were extended to non-university towns in England (Jones, 1981, 1997). Agricultural extension in Europe started with industrialization and the independence of farmers while it emerged in the USA at the same time with the broad westward migration of settlers to new and unfamiliar regions who were in need of research, training and advice about the farming conditions of the new sites (Jones, 1981).

Several definitions of ‘agricultural extension’ are available and, depending on the cultural evolution of a region or country, either participatory or rather top-down relations between the advisor and the clients exist (Knorr *et al.*, 2007). Purcell (1994), for example, understands ‘Agricultural Extension as a process of assisting farmers to become aware of, and to adopt, improved technology from any source to enhance production efficiency, income and welfare’. Contado (1997) describes both the farmers’ and the politicians’ perspectives, when defining: ‘Farmers correctly view extension as a form of assistance to help improve their know how, efficiency, productivity, profitability and contribution to the good of their family, community and society. At the same time, politicians, planners and policymakers in many developing countries view extension as a policy instrument to increase agricultural production, to achieve national food security, and, at the same time, help alleviate rural poverty’.

In many countries, the agricultural extension was at first mainly directed at male farmers, while only gradually women were included in the extension services, such as training, access to credit and in general support for income-generating activities (USAID, 2016). Extension services underwent several developments including increased participation, toward a demand-driven system (Chipeta, 2006) as well as becoming more nutrition-sensitive and including nutrition education agricultural extension (Ruel *et al.*, 2018). More emphasis on nutrition education arose in the USA and other countries to alleviate nutrition and health problems in rural areas while most recently the ‘One Health’ approach combines human and veterinary medicine to achieve optimal health—including optimal nutrition—for people, domestic animals, wildlife, plants and the environment as a whole (Lerner and Berg, 2017).

## METHODS

The study was implemented in selected sub-counties of Kakamega County in Kenya in three stages: pre-intervention (April 2016–January 2017), intervention (February 2017–September 2017) and pre-/post-evaluation (July 2016 and October 2017).

### Study design

This study piloted the approach ‘integration of nutrition messages into agricultural extension’ in rural areas and tested whether different messages and different extension systems, which communicate the nutrition topics would affect the results in terms of farmers’ KAP in relation to nutrition.

### Pre-intervention

The pre-intervention included three activities: (i) conduct training needs assessment, (ii) design and pre-test nutrition messages and (iii) build nutrition training capacity of agricultural extension workers.

The study began with three community sensitization events to inform authorities and community members. These very first meetings between researchers and community are decisive to introduce the new project, explain the upcoming activities, try to clarify any questions and seek for help with sampling of groups and participants, e.g. through providing group and household lists by the community officials. For the needs assessment, six focus group discussions (FGDs) were held in non-intervention sub-counties of Kakamega using a structured discussion guideline to identify knowledge gaps and training needs. Separate groups for women (*n* = 3) and men (*n* = 3) were created to encourage women’s participation. The average FGD group included 14 participants and FDG sessions averaged 2.5 h. Discussion topics focused on farmers’ KAP regarding nutrition, vegetable production and processing and pathways for knowledge transmission. Participant lists were provided by extension officers and local leaders based on their involvement in the vegetable value chain. Of the participants, 47% were farmers, 6% were traders and 47% were both farmers and traders. In the following, it is referred to either participants or farmers and these terms are used interchangeably.

Two types of messages were designed to communicate nutrition topics with small-scale farmers. Nutrition topics were identified from the training needs assessment which is described in more detail below. Both messages were pre-tested in non-intervention areas of Kakamega County.

A 1-day training of trainers (ToT) on delivery of the two sets of nutrition messages was conducted for the assigned Kakamega County extension officers and their cooperative partners, and the training officers from a local vegetable seed company (Simlaw Seeds). The ToT included approximately one 3 h lecture and one 3 h hands on practical and demonstrations.

### Intervention

The study intervention included three components: seed kit distribution, regular agricultural extension and nutrition communication.

#### Seed kit distribution

The seed kit consisted of five nutrient-rich traditional African vegetables: African nightshade, spiderplant, amaranth, jute mallow (*Corchorus olitorius*) and vegetable cowpea (*Vigna unguiculata*) along with a two-page leaflet explaining on how to cultivate each vegetable. The seed kits were produced by Simlaw Seeds and distributed to all groups.

#### Agricultural extension

Two agricultural extension systems in West Kenya were selected for the study: Kakamega government (public sector) and Simlaw Seeds (private sector). Two NGOs, German Agro Action and Anglican Development Service had been collaborating in Kakamega government extension activities, and we combined the public and NGO (Public/NGO) into one extension system for this study. The Public/NGO extension conducted weekly and sometimes bi-weekly group meetings. Farmer groups were trained within an existing group-based extension program focusing on agricultural production practices. The private extension provided by Simlaw Seeds trained group members on vegetable production and followed up with a total of four meetings in two crop seasons lasting 5–6 months. The two extension systems were also the two message delivery channels for nutrition communication described below.

#### Nutrition communication

Two sets of nutrition messages were integrated separately into the regular agricultural training. Both messages were based on existing nutrition communication materials (FAO, 2004, 2012; Bezner Kerr *et al.*, 2011; Voster *et al.*, 2013; ACDI/VOCA, 2016) and were tailored to the local situation with the gained information through the FGDs. These were among others the need for detailed information about food-based dietary guidelines (implemented in message 1/M1) and that available nutrition information mostly targets women’s roles only (implemented in message 2/M2). For both M1 and M2, at least 3 h of lectures and a cooking demonstration were provided along with the agricultural extension to both male and female participants (mixed groups). (M1) was developed with a focus on ‘food-based dietary guidelines’. M1 topics included basic nutrition concept, introduction of the six food groups and classifying foods into their groups, portion size of each food group, weekly plan for recipes with vegetables and general and national food-based dietary guidelines. (M2) was used to communicate explicitly with farmers of both genders. M2 topics included comparison of key stages of crop and human nutrition, benefits and challenges of crop and dietary diversity, differences in nutrient contents of various vegetables and seasonality and replaceability of nutritious foods (Table 1).

**Table 1:** Content of the two nutrition messages integrated into agriculture extension training

Nutrition message 1 (M1)	Nutrition message 2 (M2)
‘Nutrition-specific’ approach Including cooking demonstration + further hands-on participation ‘Pure’ nutrition-based message Introduction of the six food groups and classifying common foods into the six groups Daily portions of each food group that should be eaten Examples and recommendations based on a weekly plan with recipes for vegetables General food-based dietary guidelines	‘Nutrition-sensitive’ approach Including cooking demonstration Focus on integrating the nutrition message in crop production-related training sessions Comparison of four key stages of crop and human nutrition Benefits and challenges of diversifying on farm and nutritionally Differences in the nutrient content of various vegetables Seasonality and replaceability of nutritious foods

#### Sampling of participants and treatment groups

Multistage sampling was adopted to select sub-counties and villages in Kakamega county. First, 6 sub-counties out of 12 in Kakamega (Matungu, Navakholo, Malava, Mumias West, Kwisero and Ikolomani) were chosen based on high levels of malnutrition and prominence in vegetable production. Second, 2–4 villages were chosen in each sub-county, depending on the sub-county area size, for a total of 16 villages. Third, the Public/NGO and Private extensions chose one farmer group in each village assigned to them. If possible, this was a mixed gendered group, in four cases a women group took part in the study. Finally, the farmer group leaders provided a list of members, and farmers were randomly selected proportional to the size of the group for a total of 454 participants.

Three treatment groups were randomly assigned to the selected villages: the SK group received seed kits only; SK + M1 group: seed kits with the nutrition-specific message; and SK + M2 group: seed kits with the nutrition-sensitive message. The latter two are referred to as ‘intervention group’. The SK group was meant to be a control group, yet, for ethical reasons participants had to receive some input and consequently received a kind of treatment. The study is therefore a multiple treatment arm study (White, 2013). The study intervention reached 454 participants of which 375 participated in the endline survey. However, those who participated in the baseline survey were not necessarily among the 375 endline survey farmers. This is because farmers in the baseline survey were randomly selected (the third stage) from lists of all farmers in the 16 villages rather than the lists provided by farmer group leaders at the final (fourth) selection stage. Participant numbers in the baseline and endline surveys and treatment groups are listed in Supplementary Appendix Table A1.

### Pre-/post-evaluation

#### Questionnaire

The nutrition-related KAP questions were developed with reference to the FAO published ‘Guidelines for assessing nutrition-related Knowledge, Attitudes and Practices’ (Macías and Glasauer, 2015). The guidelines include 12 models of KAP questions by nutrition topic. The study questions were chosen and modified from modules 5 (under nutrition), 6 (iron deficiency), 7 (vitamin A deficiency) and 12 (food-based dietary guidelines). The questions were further modified for testing KAP in vegetable production and consumption as the agricultural extension was the major intervention in this study. The outcome variables include:

1. *Knowledge score:* measured nine knowledge-related questions with three choices: ‘Yes, I know the answer’, ‘No, I do not know the answer’ and ‘I am not sure’. The respondents answering ‘Yes’ were requested to provide a concrete answer. The interviewer decided if the answer was correct or incorrect based on the questionnaire instructions about the correctness of the answers. Responses were recorded as yes and correct (1), or yes but incorrect/no/not sure (0) and summed. The score ranged from 0 to 9.
2. *Attitude toward increased vegetable consumption*: Measured using five questions about increased belief, confidence, preference and readiness to increase family vegetable consumption. Responses were coded as positive attitude (1) or less certain/negative attitude (0). As the differentiation between positive and negative attitude was less clear than for the knowledge score we refrained from calculating an attitude score.
3. *Practices:* Two practices were measured: vegetable diversity: number of different types of vegetables grown on the farm and vegetable consumption: consumed vegetables on the previous day of the interview. Vegetable consumption was recorded using a 1-day food record.

The questionnaire asked about demographic and socioeconomic status and gardening activity. Respondent’s gender, age, education and occupation, family size, land ownership, agricultural and nutrition training received and vegetable production practices were recorded. The questionnaire was pre-tested in a Kakamega non-intervention village and modified before use in the baseline survey.

#### Data collection and analysis

Data were collected with an individual interview from the selected participants using a structured questionnaire administered by well-trained enumerators. The questionnaire was used in baseline (July 2016, *n* = 454) and endline surveys (October 2017, *n* = 375) with ∼37% of participants (*n* = 138) taking part in both. The analysis was disaggregated by message type (M1 and M2), message delivery channel (Public/NGO and Private) and pre-/post-stage. Descriptive statistics were analyzed in STATA 14. Differences between knowledge, attitude and practices of participant groups were assessed using independent *t*-test (for testing continuous variables between pre-/post-intervention) and chi-square (χ^2^) tests (for testing categorical variables between pre-/post-intervention) and results are shown in Tables 3 and 4. To test differences between the three treatment arms, an ANOVA test was applied and results are shown in the results section text. Only 37% of the participants (*n* = 138) were included in both the baseline and endline survey, which would have been a rather small panel data sample. Therefore, the difference between pre- and post-stages for KAP was simply calculated as Diff = group mean (post) − group mean (pre) for the whole sample rather than applying the difference-in-difference (DID) method which relies on panel data (Buckley and Shang, 2003).

**Table 3:** Pre-/post-results of nutrition-related knowledge by message type and delivery channel

Questions			Public/NGO		Private	
		SK	SK + M1	SK + M2	SK + M1	SK + M2
	Pre *N* =	109	113	95	72	65
	Post *N* =	129	89	81	35	41
(A) Knowledge: nutritional values of vegetables						
Q1. Do vegetables contain nutrients needed for growth and health? (%)	Pre	74.31	76.11	80.00	70.83	75.38
	Post	88.37	95.51	88.89	85.71	87.8
	Diff	14.06	19.4	8.89	14.88	12.42
	Sig.	^**^	^**^	ns	ns	ns
Q2. Do all vegetables have similar nutrient values? (%)	Pre	51.38	55.75	62.11	55.56	53.85
	Post	75.97	86.52	80.25	82.86	85.37
	Diff	24.59	30.77	18.14	27.3	31.52
	Sig.	^**^	^**^	^**^	^**^	^**^
Q3. Could you name vegetables that are high in vitamin A (or good for your eyes and protect against night blindness)? (%)	Pre	50.46	56.64	57.89	52.78	64.62
	Post	83.72	77.53	77.78	82.86	73.17
	Diff	33.26	20.89	19.89	30.08	8.55
	Sig.	^**^	^**^	^**^	^**^	ns
Q4. Could you name vegetables that are high in iron (or good for your blood and protect against anemia)? (%)	Pre	72.48	70.80	68.42	70.83	66.15
	Post	83.72	87.64	91.36	85.71	87.80
	Diff	11.24	16.84	22.94	14.88	21.65
	Sig.	^*^	^**^	^**^	ns	ns
(B) Knowledge: vegetable quantity recommended						
Q5. Do you know how many portions (handful) of vegetables you should eat each day (yes/no)? (%)	Pre	23.85	31.86	38.95	31.94	47.69
	Post	40.31	52.81	49.38	65.71	53.66
	Diff	16.46	20.95	10.43	33.77	5.97
	Sig.	^**^	^**^	ns	^**^	ns
Q6. How many portions a day? (%)	Pre	3.67	2.65	8.42	4.17	9.23
	Post	18.6	28.09	28.40	51.43	19.51
	Diff	14.93	25.44	19.98	47.26	10.28
	Sig.	^**^	^**^	^**^	^**^	ns
Q7. How much vegetable in gram per day? (%)	Pre	1.83	7.08	8.42	4.17	7.69
	Post	22.48	30.34	24.69	34.29	31.71
	Diff	20.65	23.26	16.27	30.12	24.02
	Sig.	^**^	^**^	^**^	^**^	^**^
(C) Knowledge: benefit of vegetables and vegetable diversity						
Q8. What is eating vegetables every day good for? (%)	Pre	76.15	72.57	78.95	75.00	83.08
	Post	86.82	98.88	95.06	97.14	95.12
	Diff	10.67	26.31	16.11	22.14	12.04
	Sig.	^*^	^**^	^**^	^**^	ns
Q9. Does it matter whether you eat one type of vegetable or more than 2–3 types of vegetables in a day? (%)	Pre	57.8	60.18	70.53	68.06	75.38
	Post	72.87	76.4	75.31	82.86	78.05
	Diff	15.07	16.22	4.78	14.80	2.67
	Sig.	^*^	^*^	ns	ns	ns
Overall knowledge score						
Q1—Q9 (score 0–9)	Pre	4.1	4.3	4.7	4.3	4.8
	Post	5.7	6.3	6.1	6.7	6.1
	Diff	1.6	2.0	1.4	2.4	1.3
	Sig.	^**^	^**^	^**^	^**^	^**^

Data as % of respondents providing positive answers. Diff = Post − Pre.

Sig.: ^ns^*p*-value ≥ 0.05;

* *p* < 0.05;

** *p* < 0.01.

**Table 4:** Pre-/post-results of nutrition-related attitude and practices by message type and delivery channel

Questions			Public/NGO		Private	
		SK	SK + M1	SK + M2	SK + M1	SK + M2
	Pre *N* =	109	113	95	72	65
	Post *N* =	129	89	81	35	41
Q10. How important do you think eating vegetables every day is for your family health? (% ‘very important’)	Pre	97.25	96.46	98.95	98.61	93.85
	Post	95.35	87.64	95.06	100	78.05
	Diff	−1.9	−8.82	−3.89	1.39	−15.8
	Sig.	ns	^*^	ns	ns	^*^
Q11. Do you want to increase vegetable consumption by your family? (% ‘yes’)	Pre	86.24	92.04	93.68	90.28	89.23
	Post	88.37	91.01	97.53	94.29	92.68
	Diff	2.13	−1.03	3.85	4.01	3.45
	Sig.	ns	ns	ns	ns	ns
Q12. How difficult is it for your family to eat more vegetables? (% ‘somewhat difficult’)	Pre	70.64	60.18	53.68	51.39	58.46
	Post	67.44	61.80	45.68	57.14	63.41
	Diff	−3.2	1.62	−8	5.75	4.95
	Sig.	ns	ns	ns	ns	ns
Q13. Do you have a plan how to increase consumption of vegetables in your household diet? (% ‘yes’)	Pre	65.14	80.53	81.05	75	73.85
	Post	80.62	88.76	88.89	85.71	97.56
	Diff	15.48	8.23	7.84	10.71	23.71
	Sig.	^**^	ns	ns	ns	^**^
Q14. Do you spend money on purchasing vegetables? (% ‘yes’)	Pre	76.15	81.42	82.11	86.11	86.15
	Post	71.32	64.04	72.84	77.14	39.02
	Diff	−4.83	−17.38	−9.27	−8.97	−47.13
	Sig.	ns	^**^	ns	ns	^**^
Vegetable diversity: Number of different vegetables grown on the farm	Pre	3.88	3.44	3.48	3.53	3.57
	Post	5.57	5.49	5.96	6.40	5.24
	Diff	1.69	2.05	2.48	2.87	1.67
	Sig.	^**^	^**^	^**^	^**^	^**^
Vegetable consumption: Percentage of participants who consumed vegetables on the previous day of the interview (%)	Pre	99	98	99	97	100
	Post	98	81	91	91	73
	Diff	−1	−17	−8	−0.06	−0.27
	Sig.	^**^	^**^	^*^	ns	^**^

Data were % of respondents providing positive answers. Diff = Post − Pre.

Sig.: ^ns^*p*-value ≥ 0.05;

* *p* < 0.05;

** *p* < 0.01.

## RESULTS

### Characteristics of study participants

While overall mixed farmer groups participated in the study, ∼68% of participants were female both during baseline (311 women) and endline survey (254 women). Around 15% of participants lived in female-headed households. About 50% of household heads had finished primary school, ∼30% had secondary education and 7% had no formal education (Table 2). Households consisted of about six people with a mean land size of ∼2.0 acres with 0.2 acres cropped with vegetables.

**Table 2:** Characteristics of study participants by pre-/post-surveys

Treatment group	Baseline	Endline
Number of participants	454	375
Gender HH head (% F)	14.8	16.8
Gender of participants (% F)	68.5	67.7
Education of participants		
No general education (%)	7.7	5.6
Primary (%)	52.4	49.1
Secondary (%)	31.1	34.7
Higher education (%)	8.8	10.7
Age of HH head	51 (15)	51 (13)
Age of participants	47 (14)	48 (13)
Household size	5.6 (2.1)	6.1 (2.2)
Land size (acres)	2.0 (2.3)	2.0 (2.1)

Values as an absolute number, percentage or mean value (standard deviation).

### Knowledge and communication gaps

Topics on nutrition, vegetable production and processing and knowledge transmission were covered in FGDs. We found: (i) basic nutrition concepts and food-based dietary guidelines needed reinforcement and understanding of dietary diversity for better nutrition were limited; (ii) there was little knowledge on how to prepare traditional vegetables and little interest to consume them among young people; (iii) food taboos for certain vegetables and a negative image of green leafy vegetables as ‘poor man’s crops’; (iv) available nutrition information targeted women’s roles only; (v) limited knowledge on available nutrition information sources compared to information sources on vegetable cultivation, processing and preservation. The FGD listed 24 vegetable species consumed in the county (detail in Supplementary Appendix Table A2).

### Nutrition knowledge before and after the intervention

Before the intervention, a high percentage of participants (>70% on average) knew that ‘vegetables contain nutrients needed for growth and health’ (Q1, Table 3), could name some vegetables high in iron (Q4) and recognized the importance of consuming vegetables daily (Q8). After project intervention, more participants (>85% on average) gave positive answers to these questions (Q1, Q4 and Q8). About half of participants before the intervention understood that vegetables contain different types and levels of nutrients (Q2), could name some high vitamin A vegetables (Q3) and recognized the importance of consuming diverse vegetables (e.g. 2–3 types) daily (Q9). An additional 15–30% of participants improved their knowledge for Q2, Q3 and Q9 after project intervention. Few participants (<10% on average) before project intervention were aware of the recommended quantity and portion size for daily vegetable intake (Q6 and Q7) and after the intervention participants showed significantly enhanced knowledge of daily intake amounts (Q6 and Q7) from <10% to 20–47%.

### Influence of message type on knowledge score

The nutrition knowledge score (0–9) measured the overall nutrition knowledge gains. The knowledge scores of participants receiving different messages (M1 and M2) provided by the same delivery channels were compared (Table 3). In the Public/NGO delivery channel, knowledge scores among the SK, SK + M1 and SK + M2 groups ranged from 4.1 to 4.7 before the intervention and significantly (*p* < 0.01) increased to 5.7–6.3 post-intervention. In the private delivery channel, the knowledge scores of the SK, SK + M1 and SK + M2 groups ranged from 4.1 to 4.8 before intervention and significantly (*p* < 0.01) increased to 5.7, 6.7 and 6.1, respectively, after the intervention. Results indicated that the three treatments (SK, SK + M1 and SK + M2) enhanced participants’ nutrition knowledge regardless of the delivery channel. The trend SK + M1 > SK + M2 > SK from higher to lower knowledge score was found after the intervention for both channels but was only statistically significant for the private channel (*p* < 0.01). The results implied that agricultural extension with nutrition-specific messages (M1) resulted in higher nutrition knowledge gain.

### Influence of delivery channel on knowledge score

The knowledge scores of participants receiving the same nutrition message, yet, from different delivery channels (Public/NGO and Private) were also compared (Table 3). In general, knowledge scores increased at the post-stage (*p* < 0.01) with either SK + M1 or SK + M2. A trend Private > Public/NGO > SK from higher to lower knowledge score was noted after the intervention for SK + M1, yet, the difference was not significant. The results indicate that the message delivery channel did not have a significant effect on knowledge score though private seed company training with message 1 showed higher knowledge gain.

### Attitudes toward increased vegetable consumption

At the baseline participants’ attitudes toward increasing vegetable intake was positive (Table 4). Above 90% of participants on average believed that daily vegetable consumption benefited family health (Q10, 11) and they wanted to increase consumption (Q11). More than 65% of participants planned to increase family intake of vegetables (Q13) even though many participants (52–71%) felt eating more vegetables was somewhat difficult (Q12). Around 80% of participants on average purchased vegetables.

Positive attitudes toward increasing vegetable consumption (Q10, 11) remained in the endline and somewhat less, namely 45–67% of participants, still felt eating more vegetables was challenging (Q12). More participants in the SK group (+15.5%, *p* < 0.01) and in the SK + M2 group trained by the private sector (+23.7%, *p* < 0.01) planned to increase household vegetable intake (Q13). That the study intervention reduced numbers of participants who purchased some vegetables was noted in all treatment groups and the agricultural extension activity may have led to increased home-grown vegetable production. Overall, we found high numbers of participants at baseline who believed vegetable consumption was beneficial and already wanted to increase the intake.

### Practices

Table 3 also presents the pre-/post-results of vegetable production and consumption-related practices. Crop diversity increased at endline for all treatment groups (*p* < 0.01) with the difference of increase (Diff) from higher to lower SK + M2 > SK + M1 > SK for Public/NGO channel and SK + M1 > SK + M2 > SK for Private channel. No significant difference was found among treatment groups (SK, SK + 1, SK + M2) for both delivery channels. Vegetable consumption on the day before the interview was generally high for all groups at baseline and endline (>80%), except for one group (SK + M2 from Private at endline). A reduction in the number of people consuming vegetables on the previous day was recorded at endline. Food availability and seasonality can affect food consumption, particularly for resource-poor populations. Decreased vegetable consumption at endline could be due to seasonality due to differences in data collection month for baseline in July 2016 and endline in October 2017.

## DISCUSSION

Nutrition education is defined in different ways and most definitions include a behavior change component (McNulty, 2013). This study focused on integrating nutrition communication into ongoing agricultural extension training which is increasingly practiced by more programs (FAO, 2017). While the general impact of nutrition training on farm household production and consumption was assessed before, this study compared nutrition-specific versus nutrition-sensitive messages delivered by two different channels on farmers’ knowledge and the diversity of vegetable production and consumption in smallholder households.

Some nutrition knowledge gaps identified in the FGDs could have been addressed by general nutrition education for which material is already available [e.g. (FAO, 2004)]. Nevertheless, the best training content takes into account regional differences, economic levels, cultural beliefs and other local factors. One major challenge found during FGDs was that available nutrition information mostly targets women’s roles only. In fact, there is a lack of nutrition messages that are not explicitly targeted at women as is often the case, for example, messages on infant and young child feeding, complementary feeding and breastfeeding (FAO, 2018). While to the best of our knowledge there are no explicit studies on nutrition education for men in East Africa, it is well known that male-headed households have in general higher dietary diversity (Ochieng *et al.*, 2018) and that men’s dietary knowledge can have a positive effect on the dietary diversity of women, children and the whole household (Ambikapathi *et al.*, 2021). Similar to the latter study from Ethiopia we also suggest that men need to be involved in behavior change communication activities regarding dietary diversity and nutrition knowledge more strongly as this offers great potential to improve households’ nutrition.

Interestingly, the SK group participants who received seed kits without nutrition communication showed significantly increased knowledge at the endline, although the increased score was less than the other two groups who received agricultural training and different nutrition messages. Seed kit availability probably enabled farmers in the SK group to increase vegetable production and possibly increase vegetable consumption. In addition, the baseline and endline interviews containing several questions about vegetables and nutrition may have sensitized the SK group to this topic and they may have gained information through the surveys. Other training, partly also on nutrition, took place during the study intervention period in study villages and knowledge sharing and distributing new seeds/crops were possible (Stewart *et al.*, 2015).

Compared to the SK group, the intervention groups (SK + M1 and SK + M2) showed improvement in knowledge on the numbers of vegetable portions to be eaten daily. This knowledge is important in household food selection and consumption of a balanced diet, and nutrition training seems to be especially important in conveying messages on recommended amounts of foods. Significant differences between the two nutrition messages were mainly seen for the sections on ‘vegetable quantity recommended’ and ‘benefits of vegetables and vegetable diversity’. The most obvious difference between message types was on knowledge of the number of portions of vegetables to be eaten daily, which was stronger in the nutrition-sensitive message (M2), although only for the private company participants.

Regarding participant acceptance of the training, some trainers who carried out the ToT before the intervention reported that M1 was well received by farmers since it involved direct nutrition messages. It is important to note that M1 training had much hands-on participation including sticking food pictures onto appropriate food groups and weighing different foods based on the nutrition requirement quantities to better visualize the required portion sizes. Obviously, the hands-on training enabled farmers to take up nutrition messages more easily. This kind of participatory nutrition training was also highly successful in previous studies (Waswa *et al.*, 2015) and together with participatory agricultural interventions can significantly improve child growth (Bezner Kerr *et al.*, 2011) or be integrated successfully in larger agriculture platforms (Kadiyala *et al.*, 2016). In comparison, it was slightly more difficult for farmers to relate M2 directly with nutrition. Starting with crop production was not necessarily helpful to better understand human nutrition issues and the production-nutrition connection was not easily communicated according to the trainers. However, this was only true for the private company training where M1 training yielded higher knowledge scores than M2 training. Still, the more hands-on training (M1) as a participatory communication strategy is preferable to the M2 training, yet, a hands-on training part could be also integrated into the M2 approach. So far to the best of our knowledge, there is no review study to compare different nutrition education approaches integrated into agriculture interventions or platforms in developing countries and in general a clear definition of different approaches of ‘nutrition education’ and ‘nutrition training’ in these environments is needed.

Nutrition-sensitive training was tested in Ethiopia by Agricultural Cooperative Development International and Volunteers in Overseas Cooperative Assistance (ACDI/VOCA) and showed that awareness of nutrition and dietary diversity increased among participants and that household dietary diversity level improved as well as good agricultural and hygiene practices (ACDI/VOCA, 2016). An important step in our study was to tailor this training to the local context as was reported from other nutrition-sensitive agriculture interventions (Kumar *et al.*, 2018). In this study, we found that direct nutrition-specific training, even when targeting both men and women farmers might be more effective. However, the naming of the nutrition approaches should not be confused with ‘nutrition-sensitive agriculture’ interventions which, for example, add nutrition training (of a different kind) to agriculture activities and have shown to be effective in improving nutrition (Carletto *et al.*, 2015), although a successful behavior change communication in the context of agriculture programs is usually time and resource-intensive (Ruel *et al.*, 2018).

Our results offer evidence that seed companies could capably integrate nutrition messages into their training programs and deliver favorable outcomes on par with the Public/NGO. Knowledge dissemination should, therefore, not only be channeled through public extension sectors here in combination with NGOs, and the private agricultural sector should be also considered as knowledge vehicles for nutrition topics. Although Simlaw Seeds was a project partner, it was selected because it was interested in nutrition and already promoting the nutritional values of African traditional vegetables. Because many local seed companies have extensive contacts with farmers through marketing and technical networks and through promotional events, such as field days, they could potentially be important allies in the spread of nutrition messages in rural areas. Most seed companies, however, do not presently promote nutrition but might do so if convinced that increased sales of more and diverse types of vegetable seeds could follow from increased demand for vegetables. As mentioned before, the teaching of accurate nutrition information should be the focus while critically evaluating the seed companies profit motive as they might naturally place increased importance on rising sales of seeds and other inputs (Flachs and Stone, 2019).

For logistical reasons the baseline and endline surveys were carried out in different months, namely July (dry season) and October (wet season), coinciding with different seasons complicating interpretation of the results of treatment impacts on vegetable production and consumption. Vegetable diversity grown by farmers was lower at baseline and higher at endline for the SK group and nearly the same as the intervention groups. Seed availability is often a constraint for farmers and seed provided through the project likely enabled some interested farmers to grow vegetables. Participants purchased more vegetables during baseline than during endline (SK + M1 group) and the availability of vegetable seeds may have allowed farmers to grow more vegetables at endline and reduce vegetable purchases.

### Study limitations

The study design was meant to use a quasi-experiment, eventually, we were not able to do so. The farmers randomly selected at the village level for the baseline survey could not be randomly assigned to treatment and comparison groups due to the limitation of ongoing extensions, which were an ‘in-kind’ project activity. Farmers participating in the intervention and endline survey were selected from narrowed-down lists of target farmers provided by the public and private extensions, and only 37% of the study participants took part in both baseline and endline surveys. Thus, we compared the changes with the difference between pre-/post-group means instead of the difference between pre-/post-participant data. Applying DID analysis and using participant data would have been more powerful and convincing. However, the baseline data covering the 16 selected villages from six different sub-counties out of twelve remain representative and valid and comparing pre-/post-group means were also meaningful.

Several agriculture and health-related projects and extensions were ongoing in western Kenya and these could have influenced study results. Other trainings received were monitored and, indeed, the study found increased numbers of participants received other trainings during the study period and 16% of participants said these programs included nutrition topics. This could have partially contributed to the positive nutrition knowledge gains found in this study. However, the positive effects due to the study intervention on farmers’ nutrition knowledge measured by each knowledge-related question or overall knowledge score are obvious with statistical significance.

### Study implications

We found that (i) nutrition-specific message communication in regard to consumption, even when targeting farmers of both sexes and not just those directly responsible for household food preparation, was more effective than a nutrition-sensitive message communication; (ii) nutrition knowledge can be channeled through public extension and motivated private companies; (iii) nutrition communication is especially important in conveying recommended amounts of vegetables, and other food groups; (iv) distribution of vegetable seed kits alone showed positive effects on nutrition knowledge but the combination of seed kit distribution and agricultural and nutrition training was more effective.

## SUPPLEMENTARY MATERIAL

Supplementary material is available at *Health Promotion International* online.

## Supplementary Material

daab142_Supplementary_Data_Appendix

## References

[daab142-B1] ACDI/VOCA (2016) Nutrition-Sensitive Agriculture Farmer Training. Training-of-Trainers Facilitator’s Guide. Washington, DC. http://www.acdivoca.org/wp-content/uploads/2016/06/Nutrition-sensitive-ag-training-guide-4-16.pdf (4 October 2019, date last accessed).

[daab142-B2] Aguirre T. , Hudson D. B., Weber K., Pozehl B., Boeckner L., Wilhelm S. (2012) Mexican American mothers’ eating and child feeding behaviors. Issues in Comprehensive Pediatric Nursing, 35, 4–23. 22250964 10.3109/01460862.2012.646462

[daab142-B3] Ambikapathi R. , Passarelli S., Madzorera I., Canavan C. R., Noor R. A., Abdelmenan S. et al (2021) Men's nutrition knowledge is important for women's and children's nutrition in Ethiopia. Maternal and Child Nutrition, 17, e13062. 32755057 10.1111/mcn.13062 PMC7729551

[daab142-B4] Bezner Kerr R. , Berti P. R., Shumba L. (2011) Effects of a participatory agriculture and nutrition education project on child growth in northern Malawi. Public Health Nutrition, 14, 1466–1472. 21059284 10.1017/S1368980010002545

[daab142-B5] Buckley J. , Shang Y. (2003) Estimating policy and program effects with observational data: the “differences-in-differences” estimator. Practical Assessment, Research & Evaluation, 8, 24.

[daab142-B42] Carletto G. , Ruel M., Winters P., Zezza A. (2015) Farm- Level Pathways to Improved Nutritional Status: Introduction to the Special Issue. The Journal of Development Studies, 51, 945–957. DOI: 10.1080/00220388.2015.1018908

[daab142-B6] Chipeta S. (2006) Demand Driven Agricultural Advisory Services. NEUCHÂTEL GROUP (ed), AGRIDEA, Lindau, 30 pp.

[daab142-B7] Contado T. E. (1997) Formulating extension policy. In: FAO (ed), Improving Agricultural Extension. A Reference Manual, Rome, Food and Agriculture Organization of the United Nations, Rome, Italy. pp. 107–114.

[daab142-B8] Dickson-Spillmann M. , Siegrist M. (2011) Consumers’ knowledge of healthy diets and its correlation with dietary behaviour. Journal of Human Nutrition and Dietetics, 24, 54–60. 20880377 10.1111/j.1365-277X.2010.01124.x

[daab142-B9] FAO (2004) Family Nutrition Guide. Food and Agriculture Organization of the United Nations, Rome, Italy. http://www.fao.org/docrep/007/y5740e/y5740e00.htm (12 August 2019, date last accessed).

[daab142-B10] FAO (2012) Promoting Improved Infant and Young Child Feeding: Key Messages Book. Food and Agriculture Organization of the United Nations, Rome.

[daab142-B11] FAO (2013) The State of Food and Agriculture 2013 – Food Systems for Better Nutrition. Food and Agriculture Organization of the United Nations, Rome.

[daab142-B12] FAO (2017) Integration of Nutrition in Agriculture Extension Services in Africa. A Desk Review of Country Case Studies, Pre-Service and in-Service Training Materials. Food and Agriculture Organization of the United Nations, Accra.

[daab142-B13] FAO (2018) Nutrition – Resources and Training Materials. Food and Agriculture Organization of the United Nations, Rome. http://www.fao.org/nutrition/education/iycf/iycfresources/en/ (12 August 2019, date last accessed).

[daab142-B14] Flachs A. , Stone G. D. (2019) Farmer knowledge across the commodification spectrum: rice, cotton, and vegetables in Telangana, India. Journal of Agrarian Change, 19, 614–634.

[daab142-B15] Gillespie S. , van den Bold M., Hodge J., Herforth A. (2015) Leveraging agriculture for nutrition in South Asia and East Africa: examining the enabling environment through stakeholder perceptions. Food Security, 7, 463–477.

[daab142-B16] Gómez M. I. , Barrett C. B., Buck L. E., De Groote H., Ferris S., Gao H. O. et al (2011) Research principles for developing country food value chains. Science (New York, N.Y.), 332, 1154–1155. 10.1126/science.1202543 21636760

[daab142-B17] Jones G. E. (1981) The origins of agricultural advisory services in the nineteenth century. Social Biology and Human Affairs, 46, 89–106.

[daab142-B18] Jones G. E. , Garforth C., (1997) The history, development, and future of agricultural extension. In Swanson B. E., Bentz R. P., Sofranko A. J. (eds), Improving Agricultural Extension. A Reference Manual. FAO, Rome, pp. 3–12.

[daab142-B19] Kadiyala S. , Morgan E. H., Cyriac S., Margolies A., Roopnaraine T. (2016) Adapting agriculture platforms for nutrition: a case study of a participatory, video-based agricultural extension platform in India. PLoS One, 11, e0164002. 27736897 10.1371/journal.pone.0164002 PMC5063370

[daab142-B20] Knorr J. , Gerster-Bentaya M., Hoffmann V. (2007) The history of agricultural extension in Malawi. In Boland H., Hoffmann V., Nagel U. J. (eds), Kommunikation und Beratung. Sozialwissenschaftliche Schriften zur Landnutzung und Ländlichen Entwicklung, No. 50. Margraf Publishers, Weikersheim.

[daab142-B41] Kumar N. , Nguyen P. H., Harris J., Harvey D., Rawat R., Ruel M. T. (2018) What it takes: evidence from a nutrition- and gender-sensitive agriculture intervention in rural Zambia. Journal of Development Effectiveness, 10, 341–372. DOI: 10.1080/19439342.2018.1478874

[daab142-B21] Lartey A. (2008) Maternal and child nutrition in Sub-Saharan Africa: challenges and interventions. Proceedings of the Nutrition Society, 67, 105–108. 10.1017/S0029665108006083 18234138

[daab142-B22] Lerner H. , Berg C. A. (2017) Comparison of three holistic approaches to health: One Health, EcoHealth, and Planetary Health. Frontiers in Veterenary Sciences, 4, 163. 10.3389/fvets.2017.00163 PMC5649127 29085825

[daab142-B23] Macías Y. F. , Glasauer P. (2015) Guidelines for Assessing Nutrition-Related Knowledge, Attitudes and Practices. Food and Agriculture Organization of the United Nations, Rome. http://www.fao.org/3/i3545e/i3545e.pdf (5 August 2019, date last accessed).

[daab142-B24] McNulty J. (2013) Challenges and Issues in Nutrition Education. Background paper for the international conference on nutrition, ICN2 Second International Conference on Nutrition, Rome. http://www.fao.org/3/i3234e/i3234e.pdf (2 October 2019, date last accessed).

[daab142-B25] Njoro J. , Anthony N., de Hoogh I., Fanzo J., Chahid N., Fornah D. et al (2013) An Analysis of the Food System Landscape and Agricultural Value Chains for Nutrition: A Case Study from Sierra Leone. ICN2 Second International Conference on Nutrition, Rome. http://www.fao.org/3/a-as566e.pdf (3 July 2019, date last accessed).

[daab142-B26] Ochieng J. , Afari-Sefa V., Karanja D., Kessy R., Rajendran S., Samali S. (2018) How promoting consumption of traditional African vegetables affects household nutrition security in Tanzania. Renewable Agriculture and Food Systems, 33, 105–115.

[daab142-B27] Penny M. E. , Creed-Kanashiro H. M., Robert R. C., Narro M. R., Caulfield L. E., Black R. E. (2005) Effectiveness of an educational intervention delivered through the health services to improve nutrition in young children: a cluster-randomised controlled trial. The Lancet, 365, 1863–1872. 10.1016/S0140-6736(05)66426-4 15924983

[daab142-B28] Purcell D. (1994) Agricultural Extension. Operations Evaluation Department OED Series Lessons & Practices, No. 6. The World Bank, Wachington, DC.

[daab142-B29] Ruel M. T. , Alderman H. (2013) Nutrition-sensitive interventions and programmes: how can they help to accelerate progress in improving maternal and child nutrition? The Lancet, 382, 536–551. 10.1016/S0140-6736(13)60843-0 23746780

[daab142-B30] Ruel M. T. , Quisumbing A. R., Balagamwala M. (2018) Nutrition-sensitive agriculture: what have we learned so far? Global Food Security, 17, 128–153.

[daab142-B31] Schreinemachers P. , Patalagsa M. A., Islam M. R., Uddin M. N., Ahmad S., Biswas S. C. et al (2015) The effect of women’s home gardens on vegetable production and consumption in Bangladesh. Food Security, 7, 97–107.

[daab142-B32] Stewart R. , Langer L., Da Silva N. R., Muchiri E., Zaranyika H., Erasmus Y. et al (2015) The effects of training, innovation and new technology on African smallholder farmers’ economic outcomes and food security: a systematic review. Campbell Systematic Reviews, 11, 1–224.

[daab142-B43] USAID (2016) USAID’s legacy in agricultural development. 50 years of progress. U.S. Agency for International Development, Washington D.C.

[daab142-B33] von Grebmer K. , Saltzman A., Birol E., Wiesmann D., Prasai N., Yin S. et al (2014) Global Hunger Index: The Challenge of Hidden Hunger. Bonn; Washington, DC; Dublin. http://ebrary.ifpri.org/utils/getfile/collection/p15738coll2/id/128360/filename/128571.pdf (1 October 2019, date last accessed).

[daab142-B34] Voster H. H. , Badham J. B., Venter C. S. (2013) An introduction to the revised food-based dietary guidelines for South Africa. South African Journal of Clinical Nutrition, 26, S5–S12.

[daab142-B35] Wardle J. , Parmenter K., Waller J. (2000) Nutrition knowledge and food intake. Appetite, 34, 269–275. 10888290 10.1006/appe.1999.0311

[daab142-B36] Waswa L. M. , Jordan I., Herrmann J., Krawinkel M. B., Keding G. B. (2015) Community-based educational intervention improved the diversity of complementary diets in western Kenya: results from a randomized controlled trial. Public Health Nutrition, 18, 3406–3419. 25857703 10.1017/S1368980015000920 PMC10271391

[daab142-B37] Weinberger K. , Pichop G. N. (2009) Marketing of African indigenous vegetables along urban and peri-urban supply chains in sub-Saharan Africa. In Shackleton C. M., Pasquini M. W., Drescher A. W. (eds), African Indigenous Vegetables in Urban Agriculture, 1st edition, Chapter 7. Earthscan, Routledge, London, pp. 225–244.

[daab142-B38] White H. (2013) An introduction to the use of randomised control trials to evaluate development interventions. Journal of Development Effectiveness, 5, 30–49.

[daab142-B39] WHO (2018) Healthy Diets. World Health Organization of the United Nations, Geneva. https://www.who.int/news-room/fact-sheets/detail/healthy-diet (10 June 2019, date last accessed).

[daab142-B40] Yang R.-Y. , Keding G. B. (2009) Nutritional contributions of important African indigenous vegetables. In Shackleton C. M., Pasquini M. W., Drescher A. W. (eds), African Indigenous Vegetables in Urban Agriculture, 1st edition, Chapter 4. Earthscan, Routledge, London, pp. 105–143.

