# Supplementary material for: Nutrition integrated agricultural extension—a case study in Western Kenya
Source: Health Promot Int. 2021 Sep 7;37(2):daab142. doi: 10.1093/heapro/daab142 (PMC9053460; doi:10.1093/heapro/daab142)
Supplement: daab142_Supplementary_Data_Appendix [file daab142_supplementary_data_appendix.docx]

**Appendix**

**Table A1:** Number of farmers participated in the study (treatment group, baseline and endline surveys)

| **Treatment group** | **SK** | **SK+M1** | **SK+M2** | **Total** |
| --- | --- | --- | --- | --- |
| No. of farmers selected at village level for baseline survey | 109 | 185 | 160 | 454 |
| No. of farmers selected at farmer group level, and participated in the treatment groups | 151 | 151 | 152 | 454 |
| No. of farmers that participated in the treatment groups and the endline survey | 129 | 124 | 122 | 375 |
| No. of farmers that participated in both surveys | 42 | 33 | 63 | 138 |
| No. of villages/ farmer groups | 4 | 6 | 6 | 16 |

**Table A2**: Vegetable species in Kakamega County and family members who prefer to or do not eat them for different reasons

| **English name** | **Local name** | **Scientific name** | **Family members who preferably eat the vegetable** | **Reason** | **Family members who do NOT eat the vegetable** | **Reason** |
| --- | --- | --- | --- | --- | --- | --- |
| Jute mallow leaves | Mrenda | *Corchorus olitorius* | Pregnant women  Lactating mothers  Women and children  All family members  Men of some clans | For ease of delivery Stimulates milk production  Cheap and strengthens joints and bones  Purifies the chest/good for asthma patients  Adds sexual energy and general body energy | Men  Some clans e.g., Basoi (Local herbalists) | Do not like it because of the slimy texture.  They believe if eaten a curse will reach its target and herbs won’t heal |
| Malabar spinach | Nderema | *Basella alba* | Lactating mothers | Adds blood and stimulates milk production | Children | Do not like it because of the bitterness |
| Pumpkin leaves | Seveve | *Cucurbita* sp. | Those with weak immunity | Promotes blood formation | Some clans | They swore and believed they cannot eat it. Causes itching if eaten.  It is food for snakes |
| Arrowroot leaves  Cassava leaves  Bean leaves  Mushrooms | Makalava  Shirietso Matere | *Maranta* sp.  *Manihot esculentus*  *Phaseolus vulgaris*  *Erythrococca bongensis* | Old family members | They know how to prepare it | - | - |
| Bean leaves | Makalava | *Phaseolus vulgaris* | - | - | Some clans | They swore never to eat |
| Crotalaria, rattlepod | Miroo | *Crotalaria* sp. | - | - | Some families | They believe if eaten in plenty it can cause kidney problems |
| Crotalaria  African nightshade | Miroo  Lisutsa | *Crotalaria* sp.  *Solanum* sp. | - | - | Young men and women | They do not like the taste of the bitter varieties |
| Cowpea leaves | Kunde | *Vigna unguiculata* | - | - | Lactating mothers | Stops milk (supply) |
|  | Shirietso | *Erythrococca bongensis* | - | - | Lactating mothers Some clans  The young generation | Stops milk (supply)  Belief that consumption causes insanity  They do not know how to prepare it |
| Sweet potato leaves | Milavi | *Ipomoea batatas* | - | - | Some members | Lack information on preparation |
|  | Imbetsa  Shirietso Murunde Linyolonyolo | *Erythrococca bongensis* | - | - | Some families | Associated with the low class in the society |
